# The Role of Extracellular Vesicles in Disease Progression and Detection of Hepatocellular Carcinoma

**DOI:** 10.3390/cancers13123076

**Published:** 2021-06-20

**Authors:** Yi-Te Lee, Benjamin V. Tran, Jasmine J. Wang, Icy Y. Liang, Sungyong You, Yazhen Zhu, Vatche G. Agopian, Hsian-Rong Tseng, Ju Dong Yang

**Affiliations:** 1 California NanoSystems Institute, Crump Institute for Molecular Imaging, University of California, Los Angeles, CA 90095, USA; Yi-TeLee@mednet.ucla.edu (Y.-T.L.); icybingliang@gmail.com (I.Y.L.); YazhenZhu@mednet.ucla.edu (Y.Z.); HRTseng@mednet.ucla.edu (H.-R.T.); 2 Department of Molecular and Medical Pharmacology, University of California, Los Angeles, CA 90095, USA; Jasmine.Wang@cshs.org; 3 Department of Surgery, University of California, Los Angeles, CA 90095, USA; BVTran@mednet.ucla.edu (B.V.T.); VAgopian@mednet.ucla.edu (V.G.A.); 4 Jonsson Comprehensive Cancer Center, University of California, Los Angeles, CA 90048, USA; 5 Samuel Oschin Comprehensive Cancer Institute, Cedars-Sinai Medical Center, Los Angeles, CA 90048, USA; Sungyong.You@cshs.org; 6 Division of Cancer Biology and Therapeutics, Departments of Surgery, Cedars-Sinai Medical Center, Los Angeles, CA 90048, USA; 7 Karsh Division of Gastroenterology and Hepatology, Cedars-Sinai Medical Center, Los Angeles, CA 90048, USA; 8 Comprehensive Transplant Center Cedars-Sinai Medical Center, Los Angeles, CA 90048, USA

**Keywords:** hepatocellular carcinoma, extracellular vesicles, disease progression, cancer detection, biomarker, liquid biopsy

## Abstract

**Simple Summary:**

Extracellular vesicles (EVs) are particles naturally released from cells and mediate intercellular communication. Recently, emerging studies have shown that EVs play a crucial role in regulating progression of hepatocellular carcinoma (HCC), which is one of the leading causes of cancer-related death worldwide. With the advances of technologies in isolating EVs from patients’ blood, EVs are regarded as promising biomarkers for detecting HCC at an earlier stage. This review provides an overview of the current EVs isolation methods, the biological roles of EVs in mediating disease progression, and the feasibility of EVs’ use for detection of HCC.

**Abstract:**

Hepatocellular carcinoma (HCC) is the most common primary liver malignancy and one of the leading causes of cancer-related death worldwide. Despite the improvements in surveillance and treatment, the prognosis of HCC remains poor. Extracellular vesicles (EVs) are a heterogeneous group of phospholipid bilayer-enclosed particles circulating in the bloodstream and mediating intercellular communication. Emerging studies have shown that EVs play a crucial role in regulating the proliferation, immune escape, and metastasis of HCC. In addition, because EVs are present in the circulation at relatively early stages of disease, they are getting attention as an attractive biomarker for HCC detection. Over the past decade, dedicated efforts have been made to isolate EVs more efficiently and make them useful tools in different clinical settings. In this review article, we provide an overview of the EVs isolation methods and highlight the role of EVs as mediators in the pathogenesis and progression of HCC. Lastly, we summarize the potential applications of EVs in early-stage HCC detection.

## 1. Introduction

Hepatocellular carcinoma (HCC) is the most common primary liver malignancy (>80% cases) and ranks sixth for cancer incidence and third for cancer-related death worldwide [1,2]. Main risk factors for hepatocarcinogenesis include chronic hepatitis B virus (HBV) and hepatitis C virus (HCV) infection, fatty liver disease and diabetes, alcohol consumption, and liver cirrhosis caused by any disease [2]. Despite the improvements in surveillance programs and treatment algorithms, the overall survival of patients with HCC remains dismal, with a 5-year overall survival rate of approximately 20% [3]. In light of this, biomarkers that could sensitively detect early-stage HCC have been under investigation [4]. In parallel, significant research efforts are undergoing to identify the mechanisms involved in HCC pathogenesis to prevent tumor progression and metastasis [5].

Extracellular vesicles (EVs) are a heterogeneous group of phospholipid bilayer-enclosed particles that are released by both tumor and normal cells [6]. Among the three major tumor liquid biopsy approaches, i.e., circulating tumor DNA [7], circulating tumor cells [8,9], and EVs [10], EVs are present in circulation at relatively early stages of disease and persist across all its stages [11]. Furthermore, the quantity of EVs is 2.3- to 3.0-fold higher in HCC cases than in cirrhosis controls [12,13], making them ideal biomarkers for non-invasive diagnosis of liver cancer. In addition, EVs are crucial mediators of cell-to-cell communications through transfer of functional cargoes from one cell to another [11]. As such, profiling the distinctive compositions of proteins, lipids, and nucleic acid in EVs may provide valuable information for understanding the pathological process of cancer.

Over the past decade, there have been emerging studies investigating the roles of EVs in HCC tumorigenesis. In this review, we summarize the EVs detection technology, role of EVs in cancer cell proliferation, angiogenesis, and metastasis of HCC, and the feasibility of EVs’ use as a diagnostic biomarker for HCC.

## 2. Nomenclature

Endorsed by the International Society for Extracellular Vesicles (ISEV), EVs are defined as lipid bilayer-delimited particles naturally released from cells that do not contain a functional nucleus for replication [6]. EVs are present in almost all the main body fluids and tissues and contain proteins, lipids, DNAs, messenger RNAs (mRNAs), microRNAs (miRNAs), and other non-coding RNAs, such as circular RNAs (circRNAs) and long non-coding RNAs (lncRNA) for cell-to-cell communication [11]. Historically, EVs are categorized into several subtypes, such as exomeres (size: ~35 nm), exosomes (size: 60–20 nm), microvesicles (size: 100 nm–1 µm), and large oncosomes (size: 1–10 µm), by size and biogenesis [14]. However, according to Minimal Information for Studies of Extracellular Vesicles 2018 (MISEV 2018) guidelines, these formerly used terms should be avoided due to their inaccurate definitions of size or biogenesis [6]. Currently, it is recommended to classify EVs by (1) physical characteristics, such as size or density with specific ranges defined, e.g., <200 nm (small EVs), or >200 nm (medium/large EVs); (2) biochemical and molecular components, e.g., epithelial cell adhesion molecule (EpCAM)^+^/CD147^+^ EVs; and (3) descriptions of conditions or cell of origin, e.g., HCC-derived EVs [6].

After being released from parental cells, EVs migrate to recipient cells for subsequent intercellular communication [11]. Numerous studies have shown that EVs are involved in the pathogenesis, differentiation, proliferation, and metastasis of HCC. Investigation of the molecular contents in EVs from different disease states would therefore be helpful to understand the landscape of HCC.

## 3. EVs isolation method

**Figure 1:**
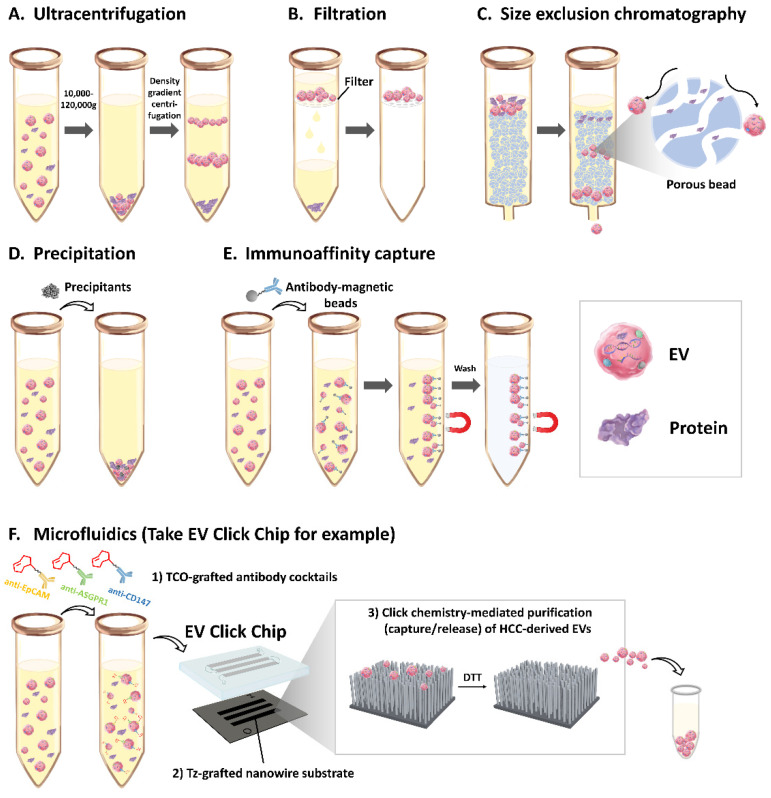
Schematic summary of the EVs isolation methods. (**A**) Ultracentrifugation. By centrifugation at the speeds of 10,000–20,000 g and 100,000–120,000 g, large and small EVs are separated, respectively. Density gradient ultracentrifugation can be used for further EVs purification. (**B**) Filtration. Using a series of membrane filters with different pore diameters enables isolation of EVs with a specified size. (**C**) Size exclusion chromatography. The chromatography column consists of porous beads only allowing smaller particles, such as proteins, to enter. EVs are larger than the pore size; therefore, they migrate at a higher speed than the smaller particles and are isolated. (**D**) Precipitation. Precipitants occupy the solution and make less soluble particles, including EVs, exceed their solubility limit and precipitate. (**E**) Immunoaffinity capture. Antibodies targeting surface proteins on EVs are used to positively or negatively select specific subpopulations of EVs. (**F**) Microfluidics. For example, EV Click Chip: (1) the multimarker antibody cocktails enable targeting HCC-derived EVs, (2) nanostructured substrates increase the surface interacting with EVs, and (3) click chemistry-mediated EVs capture (TCO/TZ interaction) and disulfide cleavage lead to DTT-driven EVs release, which results in isolation of HCC-derived EVs with high purity. ASGPR1, asialoglycoprotein receptor 1; DTT, 1,4-dithiothreitol; EpCAM, epithelial cell adhesion molecule; EVs, extracellular vesicles; HCC, hepatocellular carcinoma; TCO, trans-cyclooctene; Tz, tetrazine.

In general, there are at least six different types of EVs Isolation Methods (Figure 1): (1) ultracentrifugation, (2) filtration, (3) size exclusion chromatography, (4) precipitation, (5) immunoaffinity capture, and (6) microfluidics. There is no single optimal method for EVs isolation [6]. These methods can be used either alone or in combination to achieve the desired recovery and purity for downstream applications.

### 3.1. Ultracentrifugation

Ultracentrifugation is the most commonly used method for EVs isolation [15]. EVs are isolated by sequential centrifugations at increasing centrifugal forces, based on size and density. Using low-speed centrifugation, cells, platelets, apoptotic bodies, and cell debris are first eliminated from the EVs. Larger EVs and smaller EVs are then separated commonly at the speeds of 10,000–20,000 g and 100,000–120,000 g, respectively [16,17,18]. During ultracentrifugation, contaminants, including protein aggregates and lipoprotein particles, are also sedimented [17,18]. Additional steps of density gradient ultracentrifugation could be adopted to further purify the EVs [19,20,21]. However, ultracentrifugation is time-consuming and laborious and thus is unlikely to be applicable for routine use in clinical applications [16,17,18].

### 3.2. Filtration

Size-based separation of EVs from other non-EV particles can be achieved by filtration and size exclusion chromatography. The filter allows particles smaller than the pore to pass through while particles larger than the pore remain on the filter. Using a series of membrane filters with different pore diameters enables isolation of EVs fractions with specified sizes [22]. Filtration is amenable to clinical applications as it is less time-consuming and requires no special instrumentation. However, clogging and trapping of vesicles on the filter may lead to loss of EVs. Applying forces on particles to pass through filters can reduce the time of EVs isolation but may lead to deformation and breakup of large vesicles [23,24].

### 3.3. Size Exclusion Chromatography

Size exclusion chromatography, also known as gel filtration, separates molecules based on their size as they pass through a resin packed in a column [23,25]. The resin consists of porous beads with pores of a specific size that only allow smaller particles, such as proteins, to enter and thereby slow down their movement through the column due to increased path length. EVs are larger than the pore size and, therefore, flow through the column more quickly than soluble proteins. Therefore, the method can distinctly separate most protein contaminants from EVs [23,25]. Due to little pressure applied during the process, it preserves the structure, integrity, and biological functions of EVs [25,26,27,28]. However, there are some limitations: (1) The throughput is low, and (2) the resulting EV fraction is diluted and may require additional steps of concentration [25,29]. Some automated platforms have been developed to enable rapid and less laborious EVs isolation suitable for clinical application [30].

### 3.4. Precipitation

Precipitation of EVs in polyethylene glycol (PEG) solutions is, after ultracentrifugation, the second most common method for EVs isolation [31]. PEG is a superhydrophilic polymer, which occupies the water and concentrates other, less soluble, particles, including EVs, to the point of exceeding their solubility limit and precipitating [32]. The precipitate can then be pelleted by low-speed centrifugation [24]. Several commercial products have been developed based on precipitation to isolate EVs from biofluids, e.g., ExoQuick-TC™ Exosome Precipitation Solution (System Biosciences, Palo Alto, CA, USA), and Total Exosome Isolation Kit/Reagent (Invitrogen, Waltham, MA, USA). The method is quick, simple and allows for concurrent processing of numerous samples [31,32]. However, the major concern of this method is that non-EV proteins, such as lipoproteins, are co-precipitated along with EVs [33,34]. In addition, the presence of the polymer in purified EVs may interfere with down-stream analyses [33,34]. Therefore, this application is appropriate in samples thought to have sufficiently abundant EVs to reduce bias from contaminants. Additional steps before and after precipitation, including ultracentrifugation, filtration, or size exclusion chromatography, can be incorporated to reduce non-EV contaminants and PEG polymer [23,31].

### 3.5. Immunoaffinity Capture

Immunoaffinity capture isolates EVs based on the interaction between antibodies and surface proteins on EVs. Antibodies targeting surface proteins present on EVs are utilized to positively or negatively select specific subpopulations of EVs. These antibodies can be attached to magnetic beads [35,36,37] or microfluidic devices [38,39] (Section 3.6.), according to the desired downstream analysis. Therefore, the major benefit of this method is higher specificity and purity than those of the methods relying on physical properties [40,41]. However, selectivity can lead to lower yields [18]. Additionally, the cost of antibodies may be considerable.

### 3.6. Microfluidics

Microfluidic devices are designed as a network of microchannels to purify EVs by immunoaffinity and/or by physical characteristics [42]. Compared with traditional isolation methods, microfluidic-based isolation is more rapid (30 min–2 h), requires lower amounts of samples (50 μL–500 μL), and yields high-purity EVs in general [31,42]. However, some devices only allow low sample input and may therefore limit the applications of downstream analysis, such as massive sequencing [31,39,42,43,44]. Recently, our research team developed a microfluidic device named EVClick Chip [38], which synergistically integrates (1) multimarker antibody cocktails for targeting HCC-derived EVs, (2) nanostructured substrates for increasing the surface interacting with circulating EVs, and (3) click chemistry-mediated EVs capture and disulfide cleavage-driven EVs release, to achieve purification of HCC-derived EVs with high recovery yield (82.7%) and excellent purity (90.2%). Most importantly, we demonstrated the potential application of EV Click Chip combined with downstream reverse-transcription droplet digital polymerase chain reaction (RT-ddPCR) analysis in detection of early-stage HCC.

## 4. EVs as Mediators of Chronic Liver Disease and HCC Progression

**Figure 2:**
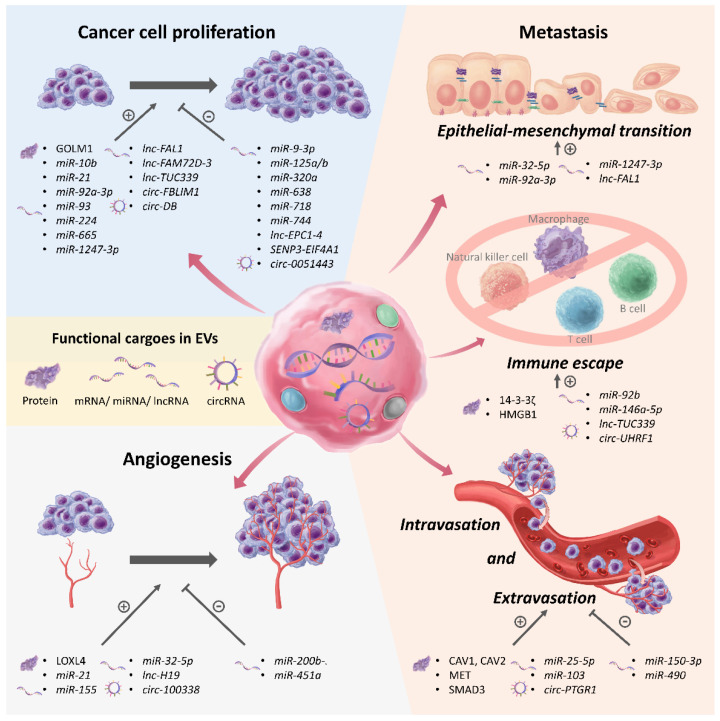
EVs as mediators in progression of HCC. Studies have demonstrated that EVs regulate cancer cell proliferation, angiogenesis, epithelial–mesenchymal transition, immune escape, and intravasation and extravasation by their functional cargoes including proteins, miRNA, lncRNA, and circRNA.

The majority of HCC diagnoses arise in patients with underlying cirrhosis, with up to 20% of HCC cases found in non-cirrhotic patients [2,45]. In a healthy liver, EVs are critical in mediating numerous signals among hepatocytes, stellate cells, and various immune cells (Kupffer cells, T and B cells, natural killer—NK—cells) to perform important functions and maintain a homeostatic state. Previous studies have demonstrated EVs play a role in the development of these predisposing liver diseases and, subsequently, in the development of HCC. EVs mediate the pathogenesis and progression process of HCC by regulating the microenvironment and multiple signaling pathways in both cancer and surrounding normal cells [46,47,48]. The studies investigating EVs’ functions in HCC progression are summarized in Table 1 and Figure 2.

### 4.1. EVs in Promoting Progression of Chronic Liver Disease

The majority of HCC cases can be attributed to chronic viral hepatitis secondary to HBV and HCV. It has been shown that inter- and intra-cellular modulation via EVs in chronic hepatitis B and hepatitis C lead to viral propagation [49,50], fibrogenesis [51,52], and dysregulation of the immune system [50,53] through various mechanisms. For example, *miR-19a* in EVs derived from HCV-infected hepatocytes initiates fibrosis by activating surrounding hepatic stellate cells through the transforming growth factor beta (TGF-β) signaling pathway [51]. In addition, studies also indicated that HBV-infected hepatocytes produce EVs to suppress the expression of interleukin (IL)-21 in T cells [53] and downregulate nuclear factor kappa B (NF-κB) in NK cells [50]. This creates a microenvironment in the cirrhotic or chronically infected liver that is primed for genetic mutations and cellular dysregulation, giving the potential for the development of HCC.

There are numerous mechanisms by which EVs promote fibrosis and inflammatory processes in metabolic liver disease. At the cellular level, fatty liver disease is caused by hepatocyte dysfunction and death due to the toxic accumulation of intra-cellular lipids and the stimulation of inflammatory and fibrogenic pathways, with EVs playing a key role [54]. Hepatocytes exposed to elevated levels of lipid, such as palmitate, had increased secretion of EVs containing tumor necrosis factor (TNF)-related apoptosis inducing ligand [55], sphingosine 1-phosphate [56], miRNAs [57,58], C-X-C motif chemokine ligand 10 (CXCL10) [59], and ceramides [60,61], leading to activation of macrophages and chemotaxis. Lipotoxic conditions also release EVs containing integrin β1, which promotes monocyte adhesion to liver sinusoidal endothelial cells, resulting in inflammation and fibrosis [62]. These findings demonstrate the roles of EVs in the progression of metabolic liver disease through inflammation and fibrosis, ultimately leading to a liver microenvironment at risk for the development of HCC.

### 4.2. EVs in Regulating Proliferation of HCC

Several studies have demonstrated that EVs could directly regulate the growth of HCC through different pathways [63,64,65,66,67,68,69,70,71,72,73,74,75,76,77,78,79]. Gai et al. found that a serum protein marker involved in the tumorigenesis and metastasis of HCC, Golgi membrane protein 1 (GOLM1), was significantly enriched in HCC-derived EVs. [63] They observed that the EV-derived GOLM1 promoted HCC proliferation, migration, and invasion and activated the glycogen synthase kinase 3β (GSK-3β)/matrix metalloproteinase-1 and -9 (MMP-1 and MMP-9) of recipient cells. miRNAs also modulate essential processes in cell proliferation at the post-transcriptional level. For example, *miR-93*, *miR-224*, and *miR-665* from HCC-derived EVs have been proven to promote HCC proliferation [67,71,72], while *miR-9-3p*, *miR-638, miR-718*, and *miR-744* have the opposite effect [64,68,69,70]. In addition to the effect of HCC-derived EVs on HCC proliferation, it was reported that EVs secreted from HCC cells promoted proliferation and suppressed apoptosis of normal hepatocytes through transferring long intergenic non-protein coding RNA, regulator of reprogramming (*linc-ROR*) [80]. After being cocultured with HCC-derived EVs for more than 30 days, the expression of stem cell-related proteins, such as OCT4, NANOG, SRY-box 2 (SOX2), P53, and CD133, in hepatocytes notably increased and these hepatic cells could still be subcultured compared with those not cocultured with HCC-derived EVs [80]. These results indicated that HCC-derived EVs-induced stem cell-like phenotype of normal hepatocytes and may lead to disease progression.

Interestingly, Tian et al. first described that an acidic tumor microenvironment, attributed to the increased glycolysis in cancer cells [81], increases the levels of *miR-10b* and *miR-21* in HCC-derived EVs compared with those produced at normal pH conditions [65]. In this study, *miR-10b* and *miR-21* were proven to promote HCC proliferation and metastasis both in vitro and in vivo. These results highlight the role of EVs in tumor progression in response to the changing microenvironment.

EVs from surrounding stromal cells, such as tumor-associated macrophages (TAMs) [82] and cancer-associated fibroblasts (CAFs) [83], participate in the regulation of HCC progression. In a recent study, Wang et al. reported a significantly lower level of *miR-125a/b* in TAM-derived EVs. Subsequent functional studies showed *miR-125a/b* in TAM-derived EVs suppress proliferation and stem cell properties of HCC in vitro [82]. Additional studies indicated that compared with EVs derived from the para-cancer fibroblasts, the CAF-derived EVs from the same HCC patients had a significantly lower level of *miR-320a* [83]. *miR-320a* in these CAF-derived EVs acts as a suppressor of HCC proliferation and migration by directly downregulating the *PBX3* oncogene.

### 4.3. EVs in Regulating Angiogenesis in HCC

In addition to tumor proliferation, EVs also modulate angiogenesis in HCC [84,85,86,87,88,89,90,91]. As a hypervascular tumor, HCC requires the formation of new blood vessels for growth. Revealing the mechanism of angiogenesis through EVs might thus help identify potential therapeutic targets to inhibit HCC progression. As proven by in vitro human umbilical vein endothelial cells (HUVECs) tube-formation assay, many molecular cargoes in EVs are key players in angiogenesis [84,85,86,87,88,89]. For example, lysyl oxidase like 4 (LOXL4) could promote angiogenesis and metastasis both in vitro and in vivo through activating the focal adhesion kinase (FAK)/Src pathway [84]. In cell line studies, *miR-155*, *lncRNA-H19*, and *circRNA-100338* from HCC-derived EVs are associated with angiogenesis [85,87,88]. On the other hand, *miR-200b-3p* and *miR-451a* suppress angiogenesis by downregulating *ERG* and *LPIN1*, respectively [86,89]. Vascular endothelial growth factor (VEGF) is a signaling protein that directly induces the growth of hepatocytes, cancer cells, and epithelial cells and leads to abnormal vascular structures in HCC. Fu et al. demonstrated that in EVs secreted from a multidrug-resistant HCC cell line, Bel/5-FU, *miR-32-5p* was the most overexpressed miRNA [90]. *miR-32-5p* raised the level of VEGF in vitro and increased the microvascular density of xenograft tumors in vivo.

### 4.4. EVs in Promoting Metastasis, Immune Escape, and Recurrence in HCC

Several molecules in EVs participate in epithelial–mesenchymal transition (EMT), extracellular matrix (ECM) remodeling, immune regulation, and cancer cell adhesion to promote HCC metastasis [66,74,75,90,92,93,94,95,96,97,98,99,100,101,102,103,104]. EMT is a process during which cells transform from a polarized, epithelial to a mesenchymal phenotype [105]. During EMT, cells lose polarity, decrease cell–cell and cell–ECM adhesions and therefore acquire increased motility and invasive properties [105]. *miR-32-5p* and *miR-92a-3p* in HCC-derived EVs suppress phosphatase and tensin homolog (PTEN) and activate the phosphoinositide 3-kinase (PI3K)/Akt (Protein kinase B, PKB) pathway to induce EMT and metastasis in vivo [66,90]. Similarly, other oncogenic proteins in HCC-derived EVs, MET and caveolins (CAV1 and CAV2), are also involved in the PI3K/Akt and mitogen-activated protein kinase (MAPK)/extracellular-signal-regulated kinase (ERK) pathways to promote migration and invasion of immortalized hepatocytes [98]. Higher expression of *miR-1247-3p* in HCC-derived EVs induces CAF activation and increases secretion of IL-6 and IL-8, thereby creating an inflammatory microenvironment [92]. Importantly, the authors demonstrated the activated CAF, in turn, further promoted EMT and metastasis of HCC in vitro and in vivo [92].

Previous studies have suggested the oncogenic roles of HCC-derived EVs in regulation of several immune cells [75,93,94,95,96,97]. The 14-3-3ζ protein is highly expressed in HCC, and impairs the anti-tumor activity of tumor-infiltrating T cells via HCC-derived EVs [93]. TAMs are one of the immune cells crucial in creation of the immunosuppressive tumor microenvironment [106]. *miR-146a-5p* and *lnc-TUC339* are enriched in HCC-derived EVs and proven to promote M2-polarization of TAMs [75,96], which can further result in T cell exhaustion [96]. In addition, Ye et al. found that the high mobility group box 1 (HMGB1) protein in EVs promoted T cell immunoglobulin and mucin domain 1 (TIM-1)^+^ regulatory B cell expansion and suppressed CD8^+^ T cell proliferation as well [94]. Lastly, it was shown that *circ-UHRF1* in HCC-derived EVs inhibited interferon gamma (IFN-γ) and TNF-α secretions from NK cells by suppressing *miR-449c-5p* and upregulating *TIM-3* [97]. All these studies provide strong evidence indicating HCC-derived EVs could induce immune escape and promote metastasis.

Tumor intravasation represents a critical step for HCC metastasis and relies on the interaction of cancer and endothelial cells [107]. Of note, Fang et al. reported that *miR-103* in HCC-derived EVs increased vascular permeability in vitro and in vivo by suppressing the expression of VE-cadherin, p120-catenin, and zonula occludens-1, which are endothelial adhesion molecules important in maintaining cell–cell junctions [101]. Interruption of the junction integrity eventually promotes liver and lung metastases. Similarly, *miRNA-25-5p* in HCC-derived EVs promotes trans-endothelial motility of HCC cells and causes tumor self-seeding in vivo [100]. Once cancer cells enter and survive in the circulation, attachment to the endothelial lining of microvasculature is essential for extravasation and consequent metastasis [108]. Fu et al. demonstrated SMAD family member 3 (SMAD3) in HCC-derived EVs-promoted adhesion of HCC cells to endothelial cells in vitro and observed a higher level of SMAD3 in EVs from patients with advanced stage HCC [99]. A non-coding RNA, *lnc-H19*, in EVs secreted from CD90^+^ HCC cells also facilitates adhesion of HCC cells to endothelium [87].

Lastly, evidence indicates EVs may be responsible for HCC recurrence after surgical treatment. By injecting HCC-derived EVs or phosphate-buffered saline (PBS) into mice of which engrafted tumors were completely resected, Chen et al. showed 100% of the mice (5/5) in the EVs injection group experienced intrahepatic recurrence, compared with the recurrence rate of only 40% (2/5) in the PBS injection group [109]. However, the molecules in EVs participating in the process of recurrence were not investigated. In another study, Nakano et al. isolated EVs from HCC patients receiving liver transplantation (LT) and demonstrated that the patients without posttransplant HCC recurrence had lower EVs-derived *miR-92b* at 1 month after LT compared with those with posttransplant HCC recurrence [95]. The authors also proved that *miR-92b* derived from HCC EVs suppresses the cytotoxicity of NK cells by, which may cause immune escape of HCC cells, followed by the induction of posttransplant recurrence [95].

**Table 1:** EVs as mediators of HCC progression.

Name of the Cargo in EVs.	Cargo Type	Level in HCC EVs ^1^	EVs isolation Method ^2^	Function of the Cargo	Mechanism of the Cargo	Ref
**HCC cell proliferation**						
GOLM1	Protein	↑	Differential ultracentrifugation	Promotes HCC cell proliferation, migration, and invasion in vitro	Activates the GSK-3β/MMP-1 and -9 pathway	[63]
*miR-9-3p*	miRNA	↓	Differential ultracentrifugation	Suppresses HCC cell proliferation in vitro	Suppresses the ERK1/2 pathway and HBGF-5 expression	[64]
*miR-10b, miR-21* (cultured at acidic condition—pH 6.6)	miRNA	↑	Differential ultracentrifugation	Promotes HCC cell proliferation, migration, and invasion in vitro; promotes HCC growth and lung metastasis in vivo	–	[65]
*miR-92a-3p*	miRNA	↑	Differential ultracentrifugation	Promotes HCC cell proliferation, migration, invasion, and EMT in vitro, promotes EMT and metastasis in vivo	Suppresses PTEN and activates the PI3K/AkT pathway	[66]
*miR-93*	miRNA	↑	Total Exosome Isolation Kit	Promotes HCC cell proliferation and invasion in vitro	Suppresses expression of TP53INP1, TIMP2, and CDKN1A	[67]
*miR-125a/b* (from TAM)	miRNA	-	ExoQuick™ Exosome Precipitation Solution	Suppresses HCC cell proliferation, migration, invasion, and stem cell properties in vitro	Suppresses CD90 expression	[82]
*miR-224*	miRNA	↑	Total Exosome Isolation Kit	Promotes HCC cell proliferation and invasion in vitro	Suppresses *GNMT* expression	[71]
*miR-320a* (from CAF)	miRNA	-	Total Exosome Isolation Kit	Suppresses HCC cells proliferation, migration and metastasis in vitro and in vivo	Suppresses the PBX3/ERK1/2/CDK2 pathway	[83]
*miR-638*	miRNA	↓	Total Exosome Isolation Kit	Suppresses HCC cell proliferation in vitro	–	[68]
*miR-665*	miRNA	↑	Differential ultracentrifugation	Promotees HCC cell proliferation in vitro, promotees HCC growth in vivo	Activates the MAPK/ERK pathway	[72]
*miR-718*	miRNA	↓	Differential ultracentrifugation	Suppresseses HCC cell proliferation in vitro	Suppresses *HOXB8* expression	[69]
*miR-744*	miRNA	↓	Differential ultracentrifugation	Suppresseses HCC cell proliferation and chemoresistance to sorafenib in vitro	Suppresses PAX2 expression	[70]
*miR-1247-3p*	miRNA	↑	Differential ultracentrifugation	Promotes proliferation of CAF in vitro, the activated CAF further promotes HCC cell progression, migration, stem cell properties, EMT, and chemoresistance to sorafenib in vitro and in vivo	Suppresses B4GALT3 to activate the NF-κB pathway	[92]
*lnc-EPC1-4*	lncRNA	↓	Differential ultracentrifugation	Suppresses HCC cell proliferation and promotes HCC cell apoptosis	–	[73]
*lnc-FAL1*	lncRNA	↑	ExoQuick-TC™ Exosome Precipitation Solution	Promotees HCC cell proliferation, migration, invasion, and EMT in vitro	Suppresses *miR-1236* to activate *ZEB1* and *AFP* expression	[74]
*lnc-FAM72D-3*	lncRNA	↑	Differential ultracentrifugation	Promotes HCC cell proliferation and suppresses HCC cell apoptosis	–	[73]
*lnc-TUC339*	lncRNA	↑	Differential ultracentrifugation	Promotes proliferation and suppresses cell adhesion to extracellular matrix of HCC cell in vitro, suppresses phagocytic activity and promotes M2-polarization of macrophage in vitro	May be involved in several pathways to regulate macrophages	[75]
*SENP3-EIF4A1*	lncRNA	↓	ExoQuick-TC™ Exosome Precipitation Solution	Suppresses HCC cell proliferation and migration in vitro, suppresses HCC growth in vivo	Suppresses *miR-9-5p* to activates *ZFP36* expression	[76]
*circ-0051443*	circRNA	↓	ExoQuick™ Exosome Precipitation Solutio	Suppresses HCC cell proliferation and promotes HCC cell apoptosis in vitro, suppresses HCC growth in vivo	Activates BAK1 expression	[77]
*circ-FBLIM1*	circRNA	↑	Differential ultracentrifugation	Promotes HCC cell proliferation and glycolysis in vitro, promotes HCC growth in vivo	Suppresses *miR-338* to activate *LRP6* expression	[78]
*circ-DB* (from adipocyte)	circRNA	-	Differential ultracentrifugation	Promotes HCC cell proliferation and reduces DNA damage in vitro, promotes HCC growth in vivo	Suppresses *miR-34a* and activates expression of *USP7* and cyclin A2	[79]
**Angiogenesis**						
LOXL4	Protein	↑	Differential ultracentrifugation	Promotes angiogenesis, HCC cell migration and invasion in vitro, promotes liver and lung metastasis in vivo	Activates the FAK/Src pathway	[84]
*miR-21*	miRNA	↑	Differential ultracentrifugation	Converts hepatic stellate cells into to cancer-associated fibroblasts and promotes angiogenesis in vitro, promotes HCC growth and angiogenesis in vivo	Suppresses PTEN and activates the PI3K/AkT pathway in hepatic stellate cells	[91]
*miR-32-5p* (from multidrug-resistant HCC cell line, Bel/5-FU)	miRNA	↑	Differential ultracentrifugation	Promotes angiogenesis, HCC cell migration, invasion, and EMT, causes multidrug resistance in vitro, promotes angiogenesis and EMT, and causes 5-FU resistance in vivo	Suppresses PTEN and activates the PI3K/Akt pathway	[90]
*miR-155* (cultured at hypoxic condition—1% O_2_)	miRNA	↑	ExoQuick-TC™ Exosome Precipitation Solution	Promotes angiogenesis in vitro	–	[85]
*miR-200b-3p*	miRNA	↓	Total Exosome Isolation Kit	Suppresses angiogenesis in vitro	Suppresses ERG expression	[86]
*miR-451a*	miRNA	↓	Differential ultracentrifugation	Suppresses cell proliferation and migration, promotes apoptosis of HCC cell and HUVEC in vitro, and suppresses angiogenesis in vitro and in vivo	Suppresses LPIN1 expression	[89]
*lnc-H19* (from CD90^+^ HCC cell)	lncRNA	↑	Differential ultracentrifugation	Promotes cell–cell adhesion of HCC cells and promotes angiogenesis in vitro	Activates VEGF expression	[87]
*circ-100338*	circRNA	↑	Differential ultracentrifugation	Promotes HCC cell invasion and angiogenesis in vitro, promotes HCC growth, angiogenesis, and lung metastasis in vivo	–	[88]
**Metastasis**						
14-3-3ζ	Protein	↑	Differential ultracentrifugation	Suppresses anti-tumor activity of tumor-infiltrating T lymphocytes	–	[93]
CAV1, CAV2, MET	Protein	↑	Differential ultracentrifugation	Promotes migration and invasion of non-motile immortalized hepatocyte cells in vitro	Activates the PI3K/AkT and MAPK/ERK pathways	[98]
SMAD3	Protein	↑	ExoQuick™ Exosome Precipitation Solution	Promotes HCC cells adhesion in vitro	Activates ROS expression	[99]
HMGB1	Protein	↑	Differential ultracentrifugation	Promotes TIM-1^+^ B cell expansion and suppresses CD8^+^ T cells activity in vitro	Activates the TLR2/4-MAPK pathway	[94]
*miR-25-5p*	miRNA	↑	Differential ultracentrifugation	Promotes transendothelial migration of HCC cell in vitro, promotes HCC tumor self-seeding in vivo	Suppresses *LRRC7* expression	[100]
*miR-92b*	miRNA	↑	ExoQuick™ Exosome Precipitation Solution	Promotes HCC cell migration and suppresses NK cells cytotoxicity in vitro	Mechanism regarding HCC migration is not mentioned Suppresses CD69 on NK cells	[95]
*miR-103*	miRNA	↑	Differential ultracentrifugation	Increases vascular permeability in vitro and in vivo, promotes liver and lung metastasis in vivo	Suppresses expression of VE-cadherin, p120-catenin, and ZO-1	[101]
*miR-146a-5p*	miRNA	↑	Differential ultracentrifugation	Promotes M2-polarization of tumor-associated macrophages and suppresses T cells activity in vitro and in vivo	–	[96]
*miR-150-3p* (from CAF)	miRNA	–	Total Exosome Isolation Reagent	Suppresses HCC cell migration and invasion in vitro	–	[102]
*miR-490* (from mast cells)	miRNA	–	Total Exosome Isolation Reagent	Suppresses HCC cell migration and invasion in vitro	Suppresses the EGFR/AkT/ERK1/2 pathway	[103]
*circ-PTGR1*	circRNA	↑	ExoQuick-TC™ Exosome Precipitation Solution	Promotees HCC cell migration and invasion in vitro, promotes mesenteric lymph node metastasis in vivo	Competes with *MET* and suppresses *miR449a* expression	[104]
*circ-UHRF1*	circRNA	↑	ExoQuick™ Exosome Precipitation Solution	Suppresses NK cell secretion of IFN-γ and TNF-α in vitro and in vivo, promotes metastasis in vivo	Suppresses *miR-449c-5p* to upregulate *TIM-3*	[97]

^1^ The label ↑ indicates that the expression level of the cargo in EVs derived from HCC cells is higher than which derived from normal hepatoctyes. The label ↓ indicates that the expression level of the cargo in EVs derived from HCC cells is lower than which derived from normal hepatoctyes. ^2^ ExoQuick™ Exosome Precipitation Solution and ExoQuick-TC™ Exosome Precipitation Solution are produced by System Biosciences, USA; Total Exosome Isolation Reagent and Kit are produced by Invitrogen, USA. 5-FU, 5-fluorouracil; Akt, AKT serine/threonine kinase 1; B4GALT3, β-1,4-galactosyltransferases III; BAK1, BCL2 antagonist/killer 1; CAF, cancer-associated fibroblasts; CAV, caveolins; CDK2, cyclin-dependent kinase 2; CDKN1A, cyclin-dependent kinase inhibitor 1A; EGFR, epidermal growth factor receptor; EMT, epithelial–mesenchymal transition; ERG, erythroblast transformation-specific-related gene; ERK, extracellular signal-regulated kinase; FAK, focal adhesion kinase; GOLM1, Golgi membrane protein 1; GSK3β, glycogen synthase kinase 3β; HBGF-5, human fibroblast growth factor 5; HCC, hepatocellular carcinoma; HUVEC, human umbilical vein endothelial cell; IFN-γ, interferon gamma; LRP6, LDL Receptor Related Protein 6; MAPK, mitogen activated protein kinase; MMP, matrix metalloproteinase; NK cell, natural killer cell; PBX2, pre-B-cell leukemia homeobox 3; PI3K, phosphatidylinositol-3 kinase; PTEN, phosphatase and tensin homolog; ROS, reactive oxygen species; TAM, tumor-associated macrophages; TIMP2, tissue inhibitor metalloproteinase-2; TLR, toll-like receptor; TNF-α, tumor necrosis factor alpha; TP53INP1, tumor protein 53-induced nuclear protein 1; USP7, ubiquitin specific peptidase 7; VE-cadherin, vascular endothelial cadherin; VEGF, vascular endothelial growth factor; ZEB1, zinc finger E-box binding homeobox 1; ZO-1, zonula occludens-1.

## 5. EVs as Biomarkers for Detection of HCC

Current tools for the detection and diagnosis of HCC include radiographic assessments (ultrasound for screening, computed tomography, and magnetic resonance imaging for diagnosis), and serum biomarkers (alpha-fetoprotein—AFP) [110]. Imaging techniques have limitations in identifying small tumors [111]. In cases of diagnostic uncertainty, invasive procedures, such as liver biopsy, may be necessary [110]. As a result of these limitations, development of novel diagnostic tools for the detection of HCC represents an unmet need. Currently available data suggest that EVs and their cargoes, including mRNAs, non-coding RNAs, and proteins, have the potential to serve as biomarkers for the detection of HCC (Table 2). These potential biomarkers can be isolated from plasma, serum, and, in some cases, bile.

Studies have demonstrated that measuring the amounts of EVs could be a strategy for the diagnosis of HCC [12,13]. By isolating EVs from peripheral blood using ultracentrifugation, Wang et al. found an increased quantity of EVs in HCC patients compared with those with liver cirrhosis [12]. The level of EVs was correlated to the tumor size and pathological classification of HCC and, most importantly, could be used to distinguishe early-stage (TNM Stage I) HCC from cirrhotic controls with an area under the curve (AUC) of 0.83 in receiver operating characteristic (ROC) analysis. Using the fluorescence-activated cell scanning, Julich-Haertel et al. identified a subgroup of EVs, the EpCAM^+^ asialoglycoprotein receptor 1^+^ (ASGPR1)^+^ EVs, which is capable of distinguishing HCC from cirrhosis with an AUC of 0.73 [13]. In both of these studies, blood samples after curative surgical treatment had significantly reduced levels of EVs, indicating the ability of these biomarkers to reflect tumor burden and monitor treatment response [12,13].

### 5.1. EV Protein for Detection of HCC

Proteomic analysis of EVs is a less explored avenue in the identification of novel biomarkers, with studies demonstrating different compositions of the proteomes of HCC, cirrhotic, and healthy control patients [112]. Arbelaiz et al. analyzed the EVs proteome profiles of intrahepatic cholangiocarcinoma (iCCA) and HCC and showed the differentially expressed proteins within these EVs could separate these two groups with an AUC of 0.89 [113]. This finding suggests EV-protein as a promising biomarker for characterizing an atypical intrahepatic lesion between HCC and iCCA. As one of the molecules mediating HCC metastasis, SMAD3, to has diagnostic power for HCC as well (AUC of 0.70 for distinguishing HCC from benign hepatoma and healthy controls) [99]. However, none of the EV-protein markers has been evaluated among at-risk patients with cirrhosis or chronic hepatitis B and patients with HCC, and further investigation is needed to assess their accuracy as a test for HCC surveillance.

### 5.2. EV miRNA for Detection of HCC

miRNAs in EVs have shown particular promise as biomarkers for the detection of HCC. Wang et al. examined the miRNA profile of EVs derived from HCC and cirrhotic patients and found that certain upregulated miRNAs (*miR-122*, *miR-148a*, and *miR-1246*) outperformered AFP in distinguishing HCC from cirrhosis. The final panel, comprising of *miR-122*, *miR-148a*, and AFP, resulted in an AUC of 0.93 [114]. It is noteworthy that the authors did not restrict the HCC cases to early-stage disease, thus likely overestimated the diagnostic power of the assay. In another study, Ghosh et al. identified four miRNAs, *miR-10b-5p*, *miR-21-5p, miR-221-3p*, and* miR-223-3p*, in liver-specific asialoglycoprotein receptor 2^+^ (ASGR2)^+^ EVs for HCC diagnosis. The combination of these four miRNAs exhibited good diagnostic power among patients with low AFP expression (<250 ng/mL), with an AUC of 0.80 [115]. Although Sohn et al. showed that clusters of miRNAs (*miR-18a*, *miR-221*, *miR-222*, *miR-224*, *miR-101*, *miR-106b*, *miR-122*, and *miR-195*) were differentially expressed among patients with chronic hepatitis B, cirrhosis and patients with HCC, no further analysis using ROC was performed to determine their diagnostic performance [116].

**Table 2:** EVs as biomarkers for detection of HCC.

Biomarkers/ Diagnostic Model	Biomarker Type	Expression Level in HCC	EV isolation Method ^1^	Number of Patients	Sen/Spe (%)	AUROC	Study Type	Restricts HCC to Early-Stage?	Ref.
Amount of total EVs	–	↑	Ultracentrifugation	28 TNM stage I HCC vs. 40 cirrhosis	63/89	0.83	Case-control	Yes	[12]
Amount of AnnexinV^+^ EpCAM^+^ ASGPR1^+^ EV	–	↑	Ultracentrifugation	86 HCC vs. 49 cirrhosis	81/47	0.73	Case-control	No	[13]
FIBG	Protein	– (↑in iCCA)	Filtration and Ultracentrifugation	29 HCC vs. 12 iCCA	83/90	0.89	Case-control	No	[113]
SMAD3	Protein	↑	ExoQuick™ Exosome Precipitation Solution	29 HCC vs. 37 HD + benign hepatoma	–/–	0.70	Case-control	No	[99]
A panel combining *miR-122*, *miR-148a*, and AFP	miRNA + AFP	↑	Ultracentrifugation, filtration, and precipitation	50 HCC vs. 40 cirrhosis	86/88	0.93	Case-control	No	[114]
A panel combining *miR-10b-5p*, *miR-221-3p*, *miR-223-3p*, and *miR-21-5p*	miRNA	↑	ExoEnrich™ instant exosome isolation kit and immunoaffinity capture (anti-ASGR2)	38 HCC vs. 35 CH + 25 cirrhosis	59/95	0.80	Case-control	No	[115]
*miR-18a, miR-101, miR-106b, miR-122, miR-195, miR-221, miR-222, miR-224*	miRNA	↑(18a, 221, 222, 224) ↓(101, 106b, 122, 195)	Ultracentrifugation	20 HCC vs. 20 cirrhosis vs. 20 CH B	–/–	–	Case-control	No	[116]
*LINC00853*	lncRNA	↑	ExoQuick™ Exosome Precipitation Solution	32 early-stage HCC (single, <2 cm) vs. 28 CH + 35 cirrhosis	94/85	0.96	Case-control	Yes	[117]
*Lnc85*	lncRNA	↑	Ribo™ Exosome Isolation Reagent	122 HCC vs. 43 cirrhosis	80/74	0.89	Case-control	No	[118]
*RN7SL1* S fragment	lncRNA	↑	Ultracentrifugation and Filtration	25 HCC vs. 25 healthy donors	–/–	0.75	Case-control	No	[119]
A risk score panel combining AFP and *ENSG00000248932.1*, *ENST00000440688.1*, *ENST00000457302.2*	lncRNA + AFP	↑	ExoQuick™ Exosome Precipitation Solution	Training set: 20 HCC vs. 20 CH Validation set: 180 HCC vs. 180 CH	–/–	0.97 0.87	Case-control	No	[120]
A panel combining *circ_0004001*, *circ_0004123*, and *circ_0075792*	circRNA	↑	Ultracentrifugation	71 HCC vs. 40 HD	91/78	0.89	Case-control	No	[121]
A panel combining 8 long RNAs	–	↑	exoRNeasy Maxi Kit	Training set: 44 HCC vs. 78 HD 1^st^ Validation set: 27 HCC vs. 53 HD 2^nd^ Validation set: 33 HCC vs. 33 HD + 6 hepatic benign disorders	84/94 89/91 –/–	0.95 0.96 0.96	Case-control	No	[122]
*LDHC*	mRNA	↑	exoRNeasy Midi Kit	50 TNM stage I/II HCC vs. 100 HD	88/93	0.95	Case-control	Yes	[123]
A panel combining *AFP*, *GPC3*, *ALB*, *APOH*, *FABP1*, *FGB*, *FGG*, *AHSG*, *RBP4*,*TF*	mRNA	↑	EV Click Chip (immunoaffinity + microfluidic device)	36 BCLC stage 0-A HCC vs. 26 cirrhosis	84/88	0.93	Case-control	Yes	[38]

^1^ ExoEnrich™ instant exosome isolation kit is produced by ExoCan Healthcare Technologies Private Limited, India; ExoQuick™ Exosome Precipitation Solution is produced by System Biosciences, USA; exoRNeasy Midi/Maxi Kit is produced by Qiagen, Germany; Ribo™ Exosome Isolation Reagent is produced by RiboBio, China. AFP, alpha-fetoprotein; AHSG, alpha 2-HS glycoprotein; ALB, albumin; APOH, apolipoprotein H; ASGPR1, asialoglycoprotein receptor 1; ASGR 2, asialoglycoprotein receptor 2; AUROC, area under the receiver operating characteristic; BCLC, Barcelona Clinic liver cancer; CH, chronic hepatitis; CH B, chronic hepatitis B; circRNA, circular RNA; FABP1, fatty acid binding protein 1; EpCAM, epithelial cell adhesion molecule; EVs, extracellular vesicles; FIBG, fibrinogen gamma chain; FGB, fibrinogen beta chain; FGG, fibrinogen gamma cha; GPC3, glypican 3; HCC, hepatocellular carcinoma; HD, healthy donors; iCCA, intrahepatic cholangiocarcinoma; LDHC, actate dehydrogenase C; lncRNA, long non-coding RNA; miRNA, microRNA; RBP4, retinol binding protein 4; RN7SL1, RNA component of signal recognition particle 7SL1; SMAD3, SMAD family member 3; TF, transferrin.

### 5.3. EV lncRNA and EV circRNA for Detection of HCC

Along with the numerous promising miRNA biomarker targets, other non-coding RNAs packaged in EVs, such as lncRNAs, have shown promise in the early detection of HCC [117,118,119,120]. One study selected six upregulated lncRNAs from The Cancer Genome Atlas by comparing 371 HCC and 50 nontumor tissues and showed that *LINC00853* in EVs was particularly promising for the identification of early-stage HCC [117]. Specifically, when setting a 14-fold increase as a cutoff for the expression of *LINC00853* in EVs, it could discriminate patients with a single, <2cm HCC from those with chronic hepatitis or liver cirrhosis, with an AUC of 0.96 [117]. By combining three EV-derived lncRNA and AFP, Lu et al. established an HCC diagnostic model and validated it in an independent validation cohort [120]. In this large validation cohort (*n* = 360), they demonstrated the model could distinguish HCC from chronic hepatitis with an AUC of 0.87 [120]. Nevertheless, failure to restrict the HCC patients to those with an early-stage disease and failure to set noncirrhotic patients as the control group are the major limitations of this study.

EV-derived circRNAs are another example of potential biomarkers for HCC detection. One study identified three upregulated circRNAs, *circ_0004001*, *circ_0004123*, and *circ_0075792*, in EVs isolated from HCC patients compared with those from healthy donors [121]. These three circRNAs are associated with the VEGF, PI3K/Akt, mechanistic target of rapamycin (mTOR), and Wnt pathways and the authors found the combination of these circRNAs had a potential for detection of HCC with an AUC of 0.89 [121]. In 2019, Li et al. performed EVs’ long RNA sequencing in five cancers including HCC to identify biomarkers for cancer diagnosis [122]. A diagnostic model containing eight long RNAs was built and validated in two cohorts to detect HCC from healthy donors and patients with unspecified benign hepatic disorders [122]. Despite the promising results with AUCs of 0.96 in the validation cohorts, there is still a concern about overestimating the diagnostic power considering the cohort composition.

### 5.4. EV mRNA for Detection of HCC

A study compared the potential of *LDHC* mRNA level in serum vs. serum-derived EVs for HCC detection. Interestingly, the authors demonstrated the superior ability of EV-derived *LDHC* mRNA to distinguish the TNM stage I/II HCC patients from healthy controls compared with serum-only *LDHC* mRNA, with an AUC of 0.95 vs. 0.84, respectively [123]. This difference might be attributed to the fact that the mRNAs in EVs are more stable than the circulating ones due to the protection by a phospholipid bilayer.

Together, all these studies demonstrate the promising application of EVs as biomarkers for the detection of HCC. It should be noted that almost all the studies enriched total EVs rather than those specifically secreted by HCC or hepatocytes. As the HCC-derived EVs represent a small portion of the total EVs, disease-specific changes in these potential EVs biomarkers may be difficult to detect, considering elevated background noise. To address this issue, our research team has developed a streamlined HCC EVs digital scoring assay [38] that couples two very powerful technologies, i.e., EV Click Chip for purification of HCC-derived EVs and RT-ddPCR for quantification of a panel of 10 HCC-specific mRNA markers. Benefiting from the nanostructured substrates, antibody cocktails—including anti-EpCAM, anti-ASGPR1, and anti-CD147—and click chemistry-mediated EVs capture/release process, EV Click Chip enables rapid and efficient purification of HCC-derived EVs. Most important of all, thanks to quantifying the 10 HCC-specific mRNA markers [124] in these purified EVs, the resulting HCC EVs digital scores exhibited promising potential for distinguishing the Barcelona Clinic liver cancer (BCLC) stage 0-A HCC from at-risk cirrhotic patients, with an AUC of 0.93 [38].

## 6. Conclusion and Future Direction

EVs play a crucial role in intercellular communication and mediate the pathogenesis, proliferation, immune escape, and metastasis of HCC [46,47,48]. As such, EVs are regarded as potential therapeutic agents or vehicles for HCC treatment [125]. With emerging studies in the field, the EVs’ cargos, including functional proteins, non-coding RNAs, and mRNAs, are promising biomarkers for the detection of early-stage HCC [126]. In parallel, dedicated efforts have been made to isolate EVs more efficiently, to facilitate the adoption of this technology for clinical applications [31,127].

Despite these encouraging results, most of the studies on detecting HCC by using EVs are still in the preclinical phase and large prospective cohort studies are warranted to validate their diagnostic value. In addition, for case-control studies to accurately estimate the diagnostic performance of EVs, it is essential to restrict the cases to early-stage HCC and the controls to at-risk patients with liver cirrhosis or chronic hepatitis B, in line with the current clinical practice guidelines which define the at-risk population to whom it is recommended to undergo screening. With the guidance of a biomarker development framework provided by the International Liver Cancer Association [128], advents of more high-quality biomarker studies on EVs for detection of early-stage HCC are expected.
